# Open Diagnostic Reader (ODR): An affordable, modular 3D-printed platform for standardized imaging and quantitative analysis of rapid diagnostic tests

**DOI:** 10.1016/j.ohx.2026.e00802

**Published:** 2026-06-05

**Authors:** Elliott Rogers, Zhen Qin, Md Taufiqur Rahman Bhuiyan, Ashraful Islam Khan, Md. Taufiqul Islam, Piyash Bhattacharjee, Imrul Kayes Nabil, Md. Naiem Hossain, Tahira Ahmed Rashmi, Moidul Hasan, Tonmoy Chandro Saha, Shawkat Osman Shishir, Mubasshir Washif, Md. Rofiqur Rahman, Imtiaz Mahamud, Maryam Shahmanesh, Taufiq Hasan, Sonia Hegde, Firdausi Qadri, Rachel A. McKendry, Andrew S. Azman

**Affiliations:** a London Centre of Nanotechnology, UCL, United Kingdom; b Institute of Global Health, UCL, United Kingdom; c International Centre for Diarrhoeal Disease Research, Bangladesh (icddr,b), Bangladesh; d Bangladesh University of Engineering and Technology (BUET), Bangladesh; e Institute for Developing Science and Health Initiatives (ideSHi) , Bangladesh; f Department of Epidemiology, Johns Hopkins University, United States; g Institute of Global Health, University of Geneva, Switzerland

**Keywords:** Rapid diagnostic tests, Imaging, Cholera, Hepatitis E, Antimicrobial resistance, Machine learning

## Abstract

Rapid diagnostic tests (RDTs) are key to disease surveillance, yet their interpretation remains challenging due to weak test lines and difficulties in interpreting results. Subjective interpretation of control and test line(s) presence and increasingly complex multiplex RDTs with many result combinations highlight the need for interpretation aides. Proprietary hardware is often expensive, fixed to specific tests, and unsuitable in decentralised testing locations. To address this, we present the Open Diagnostic Reader (ODR), a cost-effective (15 – 45 GBP), modular, easy-to-use open-source diagnostic imaging platform. The platform supports multiple lighting modalities (internal, external or no light source); multiple RDTs and disease targets (cholera and Hepatitis E); and multiple diagnostic test use cases (RDTs and agar plates for antimicrobial resistance testing). Designs can be quickly printed (19 – 25 h) using desktop 3D printers and require only simple assembly. The ODR facilitates a standardised imaging capture environment and thus reproducible image analyses pipelines, including quantitative intensity analyses and ML-enabled interpretation.

## Graphical abstract

**Figure:**
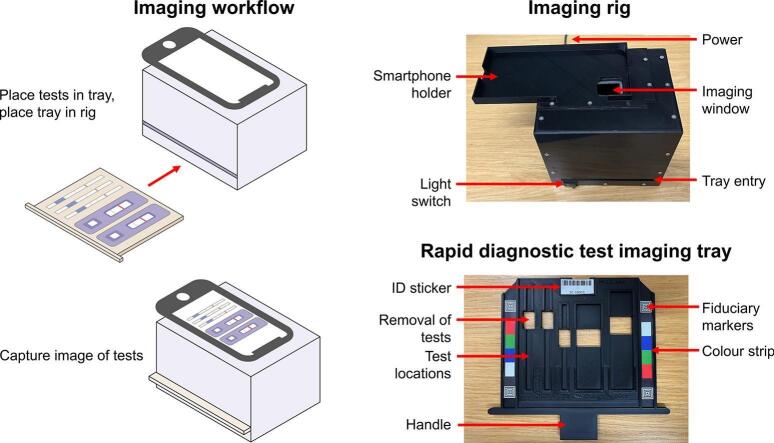

Specifications table.

**Table:** 

Hardware name	Open Diagnostic Reader (ODR)
Subject area	Engineering and materials science: Medical (e.g., pharmaceutical science)
Hardware type	Imaging tools
Closest commercial analog	SmartTester – POCT Rapid Test Reader (https://www.sterilab.co.uk/poct-rapid-test-system/smarttester)
Open source license	CC BY-SA 4.0
Cost of hardware	Imaging rig for rapid diagnostic tests with internal light source: £41.7 Imaging rig for rapid diagnostic tests with no light source: £15.4 Imaging tray for cholera rapid diagnostic tests: £3.1 Imaging tray for Hepatitis E virus (HEV) rapid diagnostic tests: £3.3 Imaging rig for agar plate with internal light source: £11.7 Imaging tray for agar plate: £4.2
Source file repository	https://doi.org/10.17632/gbzyrc9jmm.2

## 1 Hardware in context

Rapid diagnostic tests (RDTs) are low-cost diagnostics used in decentralised disease surveillance, including low-infrastructure settings in low and middle-income countries (LMIC) [1], [2], [3]. Although RDT use is increasing [3], [4], [5], [6], there remains a critical shortage of support tools for their standardised interpretation. RDTs are valued for their simple positive/negative interpretation; however, the visual interpretation of the presence of test lines is subjective. Weak test lines poorly contrasted against test strip backgrounds can lead to false positives and false negatives [7], [8], [9], [10], [11], [12], [13]. The test lines are also not permanent and can fade rapidly (usually after 15–30 mins), complicating interpretation. The WHO recently highlighted that faint positive lines, frequently seen in cases of low malaria parasitaemia, are a primary cause of false-negative reports [14], [15]. Recent longitudinal data indicate that RDTs with faint lines can be unstable, with tests reverting from positive to negative over time, and other initially negative faint-line results appearing positive later on [16]. These diagnostic inaccuracies highlight the need for standardized interpretation tools and enhanced user training. Increasing use of multiplex RDTs, such as semi-quantitative C-reactive protein (CRP) RDTs which can help combat antimicrobial resistance (AMR) [17], [18], further complicates result interpretation by requiring users to interpret multiple combinations of control and test lines.

To this end, the World Health Organisation (WHO) released a Target Product Profile (TPP) for RDT readers with software and hardware specifications. For software, machine learning (ML) interpretation of images of RDT results has been shown to improve the classification of RDTs [7], [11], [19], [20]. Meanwhile, advances in training ML models on synthetic data alongside increased mobile device market penetration, network coverage, and decreasing device costs have reduced barriers to both developing and deploying ML models to decentralised testing locations in real-time [21], [22], [23], [24]. However, the capture of real-world RDT results for classification remains non-standardized. Image capture lighting conditions, height, angle, and test location complicate interpretation. Standardized image capture is therefore essential to enable both quantitative RDT analyses, enriching the interpretation of test results beyond a binary outcome toward exploring intensity associations and quality-controlling RDTs, and automated ML-enabled interpretation of results.

The WHO TPP for RDT readers highlights the need for affordable, portable, easy-to-maintain hardware [25]. Available RDT readers can be separated into (a) those with an active light source – from expensive, standalone devices [26], [27] to cheaper, 3D printed single test readers attached to smartphone devices [28], [29] – and (b) those using natural light – from wearable glasses [30] to standalone 3D printed imaging trays [7]. Open-source hardware includes a mobile phone stand using natural light for scanning samples developed by Médecins Sans Frontières (MSF) [31].

**Fig. 1:**
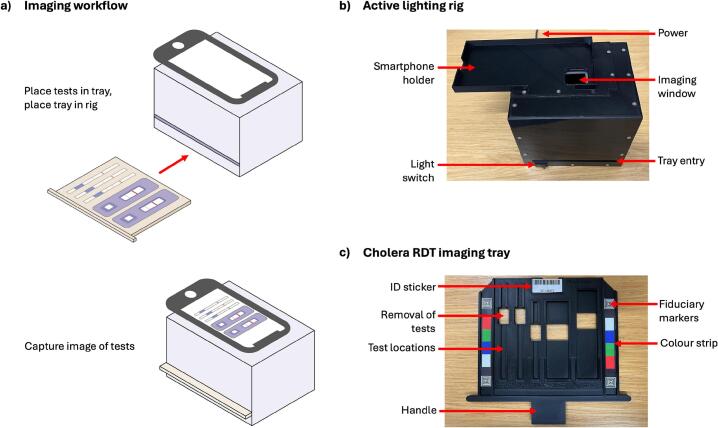
Imaging workflow (a) featuring imaging rig with an internal light source (b) and imaging tray (c). The imaging workflow illustrates placement of rapid diagnostic tests (RDTs) into the tray, placement of the tray into the imaging rig, placement of a mobile device for image capture, and the capture of images of tests. b) The imaging rig with an internal light source was designed to be cost-effective, easily assembled, easy-to-use, and highly adaptable. The rig features a universal 12 V power source, a phone holder and imaging window for the imaging device, a switch to toggle the internal LED light source, and a tray entry space to accommodate the imaging tray. c) The imaging tray was designed for five cholera RDTs, including three dipsticks (left) and two cassettes (right). The tray features a space at the top for a patient ID sticker, holes for easy removal of RDTs, fixed test locations, and a handle to manipulate the tray into the rig. The tray features angled edges for ease of entry into the rig and a wide base to block external light entering the rig. Finally, on either side of the tray, there is space for optional colour bands and fiduciary markers (high-contrast visual points) to aid with image analysis.

In this study, we present a modular, open-source 3D-printed imaging system. Our imaging platforms include an imaging tray with fixed positions for diagnostics that is placed inside an imaging rig, where a smartphone is used to capture standardized images (Fig. 1). This facilitates reproducible image analysis workflows, including traditional analysis (e.g. coordinate mapping), and automated ML segmentation and classification of regions of interest (ROIs). We further developed an imaging rig and tray to image agar plates when testing for AMR. This imaging rig uses a natural light source, reducing any glare that could be caused by an internal light source or by an external light source from the image capture device (flash). This imaging platform could standardize plate reading when combined with ML-enabled AMR diagnosis software solutions [32]. The presented imaging platforms (rig and tray) fill a critical hardware gap by providing open-source, modular, low-cost, standardized imaging platforms that can image multiple RDTs with an internal light source, and simpler imaging platforms for real-world use cases, which can be implemented alongside decentralized diagnostic use.

## 2 Hardware description

### 2.1 System architecture and modular design

**Fig. 2:**
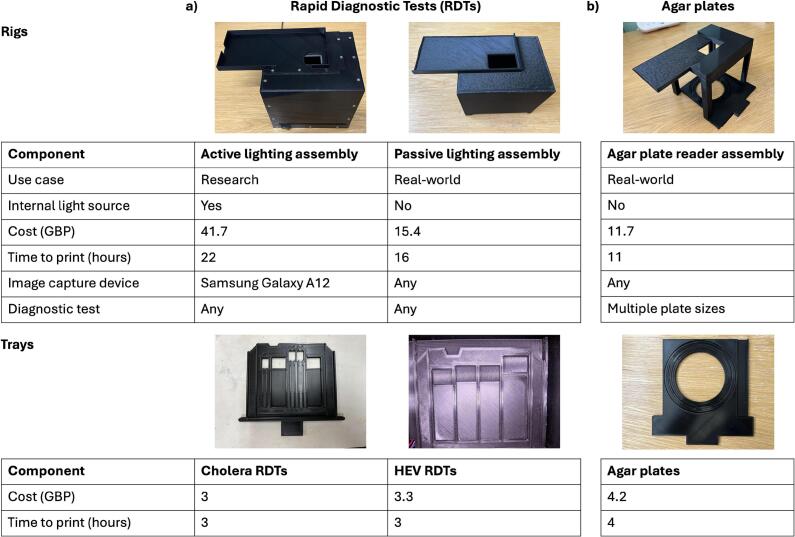
Integrated imaging platforms for multi-format diagnostic testing. Developed rigs and trays for a) Rapid Diagnostic Tests (RDTs) and b) agar plates standardize diagnostic test positions and image capture height and angle. a) Environmental conditions of RDT imaging are controlled through an internal (active lighting) or external (passive lighting) light source, enabling quantitative analysis of test line intensities. The modular design allows rigs to accommodate various tray formats, including cholera and Hepatitis E virus (HEV) RDTs. (b) The imaging rig for agar plates uses natural lighting to prevent glare that may obscure image reading. The imaging platforms are designed to be compatible with low-cost, widely available smartphones.

The Open Diagnostic Reader (ODR) platform is an open-source, modular 3D-printed optomechanical imaging platform designed to standardise the capture of diagnostic test results across diverse research and real-world use cases. Compared to the existing hardware, which are expensive, proprietary, or designed for use with individual tests, the ODR platform has a flexible architecture, as illustrated by imaging multiple tests at the same time, multiple test targets (cholera and Hepatitis E virus RDTs), multiple assay formats (RDTs and agar plates), multiple lighting configurations (supporting research and real-world use cases through internal light sources, external light sources e.g. flash, and natural light sources), and compatibility with a range of mobile devices (e.g. Samsung Galaxy A12, iPhone 13 mini). The ODR is optimized for rapid, low-cost fabrication via Fused Deposition Modelling (FDM). Our imaging platforms are cost-effective and quick to print: the active-illumination rig (rig with a light source) takes 22 h to print for 41.7 GBP; and the passive-illumination variant (rig without a light source) is printed in one piece in 16 h for 15.4 GBP. Individual trays, such as the cholera RDT imaging tray, are printed in one piece (3 h for 3.1 GBP, Ultimaker S3 3D printer) (Fig. 2, Supplementary Table 1). The ODR can be developed and printed in relatively low-tech settings, including decentralised RDT testing locations and can be used with low-cost, widely available mobile devices.

### 2.2 Design evolution and technical specifications

The ODR, including the rig and tray, was designed to meet the WHO TPP specifications for diagnostic readers, including being cost-effective, easily assembled, easy-to-use, and highly adaptable. The imaging rig with an internal light source and cholera tray was first developed for a use case of imaging multiple cholera RDTs in a research environment as part of an RDT performance evaluation. The platform underwent several iterative design cycles based on qualitative feedback from practical usage of clinical and technical personnel at the International Centre for Diarrhoeal Disease Research, Bangladesh (icddr,b) and researchers at Johns Hopkins University. Key technical refinements included:

(1) **Light management:** The focal distance between the smartphone CMOS sensor and the tray surface was optimized to ensure high-resolution capture of test lines. Additional investigated parameters included the rig width, image-capture window dimensions, and tray location and dimensions.
(2) **Adaptability:** Angled chassis guides were introduced to assist with placing the tray in the rig. A wide-base tray design created a light-tight seal, standardizing the internal environment regardless of external lighting conditions. The tray was also updated to include holes to facilitate RDT removal. Finally, the rig’s smartphone holder was adapted to accommodate different smartphones. This iterative design process illustrates the adaptability of the imaging platform.
(3) **Operational workflow:** The tray was customised for the cholera RDT imaging use case by integrating fiduciary markers and colour bands to support computer-vision analyses for quantitative test line intensity extraction. The tray also holds a patient ID sticker to aid with documenting imaging of RDTs.

### 2.3 Distributed manufacturing and global deployment

A fundamental objective of the ODR project was the decentralization of diagnostic hardware production. While initial engineering and manufacture of the RDT imaging platform with a light source was conducted at University College London (UCL), manufacturing was successfully transitioned to 3D printing enterprises in Dhaka, Bangladesh. The use of the imaging rig was expanded for use in an evaluation of Hepatitis E virus (HEV) RDTs led by the Institute for Developing Science and Health Initiatives (ideSHi), with designs shared with researchers at the Bangladesh University of Engineering and Technology (BUET). BUET researchers updated designs and localized the fabrication process of the imaging rig with a light source entirely in Bangladesh, illustrating the ease of adaptation and fabrication of the open-source designs. Designs have also been implemented in South Kivu, Democratic Republic of the Congo (DRC), to image RDTs, and further designs have been developed to image agar plates. To facilitate global accessibility, designs are made available in Tinkercad, a free web app for 3D design (Table 1).

Our Open Diagnostic Reader (ODR) features three core advantages for diagnostic research:

• Standardization: Fixed-position geometry and standardized lighting environments enable quantitative RDT intensity analysis.
• Modularity: ODR provides common interfaces with application-specific trays, enabling rapid transition between different diagnostic applications, including using different lighting settings through internal, device flashlight or natural light, or using different smartphones for image capture.
• Sustainability: Cost-effective (∼£18.5) and open-source access ensures production and integration sustainability, allowing implementers to control and easily maintain their own diagnostic infrastructure.

## 3 Design files summary

The ODR hardware is organized into three primary hardware configurations: (1) an active-illuminated rig, (2) a passive-illuminated rig (no internal lighting, flash-based), and (3) a plate-reader rig for agar plates. All CAD files are provided in Solidworks (SLDPRT) or stereolithography (STL) formats for 3D printing [33].

(1) **Active lighting assembly (rig_light_source):** A modular dark box composed of eight discrete components. The assembly includes a structural chassis (walls 1–4), a base/cover board, and a smartphone adapter with an integrated light barrier to eliminate optical glare.
(2) **Passive lighting assembly (rig_no_light_source):** A monolithic chassis optimized for single-piece printing, utilizing the image capture device's internal flash as the primary illumination source.
(3) **Rapid diagnostic test (RDT) trays:** Custom-engineered inserts for cholera (tray_cholera) and Hepatitis E (tray_hev) assays. These include calibration features, bilateral fiduciary markers for image rectification, and ergonomic handles for repeatable positioning.
(4) **Plate reader assembly:** A specialized configuration for microbiological analysis. The tray_plate interfaces with tray_plate_spacers to allow concentric alignment of varying agar plate diameters (70, 75, 80, 85, 90 mm) within the optical path of the rig_plate.

**Table 1:** Summary of hardware design files and digital repositories for the ODR components (open-source license CC BY-SA 4.0).

**Design Component**	**Design file name**	**File type**	**Location of the file**
Active Lighting Rig	rig_light_source (8 files): rig_light_source_barrier.SLDPRT rig_light_source_bottom_board.SLDPRT rig_light_source_cover_board.SLDPRT rig_light_source_side_wall_1.SLDPRT rig_light_source_side_wall_2.SLDPRT rig_light_source_side_wall_3.SLDPRT rig_light_source_side_wall_4.SLDPRT rig_light_source_smartphone_case.SLDPRT	SLDPRT, STL	SLDPRT & STL: https://doi.org/10.17632/gbzyrc9jmm.2
Passive Lighting Assembly	rig_no_light_source (1 file): rig_no_light_source.stl	STL, Tinkercad	STL: Tinkercad Link 1
Plate Reader Assembly	rig_plate (1 file): rig_plate.STL	STL, Tinkercad	STL: Tinkercad Link 2
Cholera Assay Tray	tray_cholera (1 file): tray_cholera.SLDPRT:	SLDPRT, STL	SLDPRT & STL: https://doi.org/10.17632/gbzyrc9jmm.2
Hepatitis E Tray	tray_hev (1 file): tray_hev.SLDPRT	SLDPRT, STL	SLDPRT & STL: https://doi.org/10.17632/gbzyrc9jmm.2
Agar Plate Tray	tray_plate (1 file): tray_plate.STL	STL, Tinkercad	STL: Tinkercad Link 3
Tray Spacers	tray_plate_spacers (1 file): tray_plate_spacers.STL	STL, Tinkercad	STL: Tinkercad Link 4

## 4 Bill of materials

The Open Diagnostic Reader (ODR) is designed for distributed manufacturing, utilizing 3D-printed structural components and commercially available electronic parts, when used (e.g. rig with a light source). The 3D-printed elements were fabricated using Polylactic Acid (PLA), selected for its impact resistance and dimensional stability and widespread availability across manufacturing sites in the UK and Bangladesh. Other 3D printing materials, such as Polyethylene Terephthalate Glycol-modified (PETG) or Acrylonitrile Butadiene Styrene (ABS), are likely to be equally suitable. Detailed specifications and procurement sources for the assembly are provided in Table 2.

**Table 2:** Bill of materials summary for the Open Diagnostic Reader (ODR) assembly.

**Designator**	**Component**	**Number**	**Cost per unit −currency**	**Total cost −** **currency**	**Source of materials**	**Material type**
*rig_light_source*	*Combined chassis assembly*	*308 g*	*0.07 GBP / g*	*21.30 GBP*	*https://3dgbire.com/products/ultimaker-tough-pla-black?srsltid=AfmBOorTiNro4q4_aeZPOQDisEfT6gz3AkW8KvUpV8de859-3a8b1_t0*	Polymer
*rig_light_source*	*DC-DC Non-Isolated voltage converter*	*1*	*5.11 GBP*	*5.11 GBP*	*https://www.enrgtech.co.uk/product/dc-dc-converters/ET16764261/OKL-T-1-W12N-C?srsltid=AfmBOorAhkG366W_VfDkfMaD-_Lq0NlwLw_DFfMLQk29hbRXmvBdkGbtpTs*	Other
*rig_light_source*	*Fasteners (screws)*	*27*	*5.98 GBP*	*0.20 GBP*	*https://www.amazon.co.uk/dp/B0CHB4HD8Z?psc=1&ref=ppx_yo2ov_dt_b_product_details*	Other
*rig_light_source*	*Wires*	*0.5 m*	*6.98 GBP*	*0.35 GBP*	*https://www.amazon.co.uk/dp/B07TT527TK?psc=1&ref=ppx_yo2ov_dt_b_product_details*	Other
*rig_light_source*	*12 V 1A AC/DC power supply*	*1*	*7.03 GBP*	*7.03 GBP*	*https://uk.farnell.com/pro-elec/pel00398/adapter-ac-dc-12v-1a-fixed-12w/dp/2849398*	Other
*rig_light_source*	*Tape*	*1*	*1.80 GBP*	*1.80 GBP*	*https://www.amazon.co.uk/dp/B095X9DQ1B?ref= ppx_yo2ov_dt_b_product_details&th=1*	Other
*rig_light_source*	*LEDs*	*32*	*12.99 GBP*	*2.31 GBP*	*https://www.amazon.co.uk/dp/B08T83MTGG?psc=1&ref=ppx_yo2ov_dt_b_product_details*	Other
*rig_light_source*	*Mini 2-way rocker switch*	*1*	*4.49 GBP*	*0.90 GBP*	*https://www.amazon.co.uk/dp/B00TXNXGWE?psc=1&ref=ppx_yo2ov_dt_b_product_details*	Other
*rig_light_source*	*Screw terminal plug*	*1 unit*	*6.99 GBP*	*2.33 GBP*	*https://www.amazon.co.uk/Terminal-Connector-Female-Adapter-Security/dp/B08T6NF25R*	Other
*rig_light_source*	*Light diffusion layer*	*10 cm x 20 cm*	*19.00 GBP*	*0.32 GBP*	*https://www.amazon.co.uk/dp/B08PTCGTX9?ref=ppx_yo2ov_dt_b_fed_asin_title*	Other
*rig_no_light_source.STL*	*Simplified* chassis	222 g	*0.07 GBP / g*	*15.40 GBP*	*https://3dgbire.com/products/ultimaker-tough-pla-black?srsltid=AfmBOorTiNro4q4_aeZPOQDisEfT6gz3AkW8KvUpV8de859-3a8b1_t0*	Polymer
*tray_cholera.SLDPRT*	*Cholera assay tray*	45 g	*0.07 GBP / g*	*3.10 GBP*	*https://3dgbire.com/products/ultimaker-tough-pla-black?srsltid=AfmBOorTiNro4q4_aeZPOQDisEfT6gz3AkW8KvUpV8de859-3a8b1_t0*	Polymer
*tray_hev.SLDPRT*	*Hepatitis E assay tray*	*48* g	*0.07 GBP / g*	*3.36 GBP*	*https://3dgbire.com/products/ultimaker-tough-pla-black?srsltid=AfmBOorTiNro4q4_aeZPOQDisEfT6gz3AkW8KvUpV8de859-3a8b1_t0*	Polymer
*rig_plate.STL*	*Agar plate imaging rig*	169 g	*0.07 GBP / g*	*11.70 GBP*	*https://3dgbire.com/products/ultimaker-tough-pla-black?srsltid=AfmBOorTiNro4q4_aeZPOQDisEfT6gz3AkW8KvUpV8de859-3a8b1_t0*	Polymer
*tray_plate.STL*	*Agar plate assay tray*	39 g	*0.07 GBP / g*	*2.70 GBP*	*https://3dgbire.com/products/ultimaker-tough-pla-black?srsltid=AfmBOorTiNro4q4_aeZPOQDisEfT6gz3AkW8KvUpV8de859-3a8b1_t0*	Polymer
*tray_plate_spacers.STL*	*Agar plate tray universal spacers*	22 g	*0.07 GBP / g*	*1.50 GBP*	*https://3dgbire.com/products/ultimaker-tough-pla-black?srsltid=AfmBOorTiNro4q4_aeZPOQDisEfT6gz3AkW8KvUpV8de859-3a8b1_t0*	Polymer

## 5 Build Instructions

**Fig. 3:**
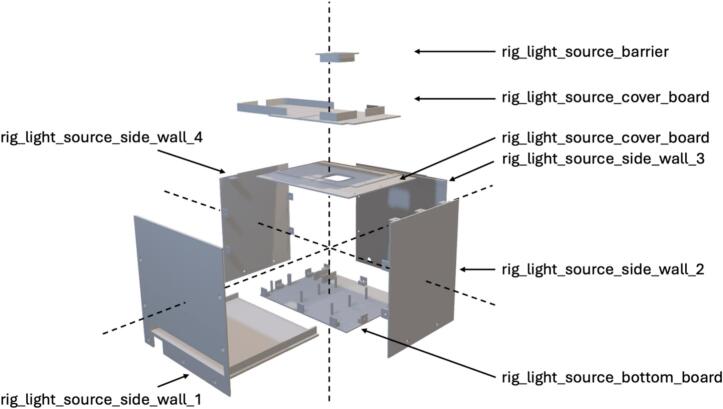
Exploded isometric diagram of the assembly of 3D printed components for the rig with a light source.

All 3D components were fabricated using an UltiMaker S3 series printer (UltiMaker, Utrecht, Netherlands) via fused filament fabrication (FFF). The primary material utilized was black UltiMaker Tough Polylactic Acid (PLA) with a filament diameter of 2.85 mm. To optimize the manufacturing process and reduce material waste, specific build orientations were selected for each component. A detailed exploded isometric diagram illustrating the assembly of the 3D-printed rig components, including the integrated light source, is provided in Fig. 3.

**Fig. 4:**
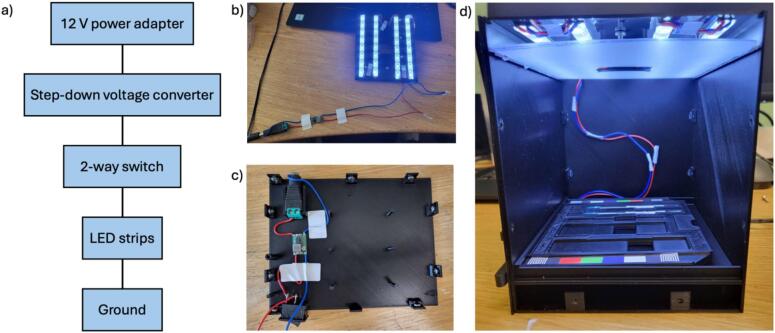
System overview and physical construction of the integrated imaging rig. (a) Electrical block diagram illustrating the power flow from the 12 V DC power adapter through the 5 V voltage converter and switch to the LED strips. (b) View of the assembled LED lighting circuit components prior to full integration. (c) Bottom view of the baseplate showing the arrangement of electronic components. (d) Fully assembled imaging enclosure with a view of the internal illumination system and imaging tray.

The fabrication of the integrated light source rig (Fig. 4) involved the assembly of eight 3D-printed components. Illumination was provided by four LED strips (eight LEDs per strip) affixed to the rig cover board (*rig_light_source_cover_board.SLDPRT*) via adhesive backing and positioned equidistantly around the camera aperture (Fig. 4b). The strips were wired in series and integrated into a circuit comprising a two-way switch, a step-down voltage converter, and a 12 V DC screw terminal connector, all mounted to the *rig_light_source_bottom_board.SLDPRT* (Fig. 4c). The structural housing was secured using three unique side walls (*rig_light_source_side_wall_1–3.SLDPRT*) fastened to the base and cover boards with screws as specified in the bill of materials (Fig. 4d). A 112 mm x 140 mm light diffusion layer, featuring a 34 mm x 34 mm aperture, was positioned 2 cm below the LEDs to allow uniform lighting illumination over the trays and further secured with adhesive tape before the final wall (*rig_light_source_side_wall_4.SLDPRT*) was attached. The assembly concluded with the mounting of the smartphone enclosure (*rig_light_source_smartphone_case.SLDPRT*) and the insertion of the light barrier into the camera aperture.

In contrast, the standard imaging rig (*rig_no_light_source.stl*), cholera and HEV RDT trays (tray_cholera.SLDPRT, tray_hev.SLDPRT), and agar plate imaging trays (tray_plate.STL and tray_plate_spacers.STL) were printed as single-piece components requiring no assembly. To optimize material efficiency and minimize support structures, specific geometries were printed in an inverted orientation with the smartphone holder interface directed toward the build plate (rig_no_light_source.STL, rig_plate.STL). Specifications of 3D prints, including print speed and infill, are available in Supplementary Table 1.

## 6 Operation instructions

Prior to operation, a comprehensive safety validation should be conducted, including testing the electrical circuit to ensure sufficient operating voltage and monitoring for excessive thermal build-up. All electrical conductors should be securely insulated, with no exposed wiring present. The imaging rig is not intended for unsupervised use or continuous operation over extended periods. Following data acquisition, all associated hardware must be cleaned and decontaminated in strict adherence to relevant infection prevention and control protocols. PLA was used as the 3D-printing medium as it is low-cost, commercially available and easy-to-print. However, it may absorb chemical or biological agents during usage, thus bringing challenges to the cleaning process. Use appropriate cleaning agents (e.g. 70% ethanol) to prevent contamination between samples. In case of imaging issues, troubleshoot the positioning of mobile device on imaging rig, positioning of tray within imaging rig, and any lighting issues (e.g. faulty LED lights, wiring issues). A video demonstrating the use of the ODR is available on the article’s Mendeley data repository.

## 7 Validation and characterization

The 3D-printed imaging rig with a light source and 3D-printed imaging tray for cholera was used daily in icddr,b labs in Dhaka, Bangladesh, for over 1 year (2024–2025), processing over 1,500 stool samples, over 17,500 cholera RDTs, and capturing over 17,500 images. Imaging was conducted using a cost-effective and widely available Samsung Galaxy A12 (Dimensions: 164.0 (L) x 75.8 (W) x 8.9 (D) mm; weight: 205 g; camera: 48MP with LED flash) [34]. The imaging rig and tray were cleaned regularly using ethanol and other decontamination materials and withstood use. The hardware has facilitated advanced image analyses of collected RDT results, including application of ML models for interpretation and ML-assisted quantitative test line intensity analyses. Due to their success, additional imaging rigs with light sources and updated imaging trays for HEV RDTs were developed and are currently deployed in HEV RDT surveillance sites, including in eight health facilities in Bangladesh. Further, newly designed imaging rigs without light sources for RDTs and agar plates are being used in South Kivu, DRC.

## Ethics statements

The authors have nothing to declare under this heading.

## CRediT authorship contribution statement

**Elliott Rogers:** Writing – review & editing, Writing – original draft, Visualization, Validation, Resources, Project administration, Methodology, Investigation, Funding acquisition, Conceptualization. **Zhen Qin:** Writing – review & editing, Validation, Methodology, Investigation, Conceptualization. **Md Taufiqur Rahman Bhuiyan:** Writing – review & editing, Validation, Investigation. **Ashraful Islam Khan:** Writing – review & editing, Validation, Investigation. **Md. Taufiqul Islam:** Writing – review & editing, Validation, Resources, Investigation. **Piyash Bhattacharjee:** Writing – review & editing, Validation, Investigation. **Imrul Kayes Nabil:** Writing – review & editing, Validation, Investigation. **Md. Naiem Hossain:** Writing – review & editing, Validation, Investigation. **Tahira Ahmed Rashmi:** Writing – review & editing, Validation, Investigation. **Moidul Hasan:** Writing – review & editing, Validation, Resources, Investigation. **Tonmoy Chandro Saha:** Writing – review & editing, Validation, Resources, Investigation. **Shawkat Osman Shishir:** Writing – review & editing, Validation, Resources, Investigation. **Mubasshir Washif:** Writing – review & editing, Validation, Resources, Investigation. **Md. Rofiqur Rahman:** Writing – review & editing, Validation, Resources, Investigation. **Imtiaz Mahamud:** Writing – review & editing, Validation, Resources, Investigation. **Maryam Shahmanesh:** Writing – review & editing, Supervision. **Taufiq Hasan:** Writing – review & editing, Validation, Supervision, Resources, Investigation. **Sonia Hegde:** Writing – review & editing, Validation, Supervision, Resources, Methodology, Investigation, Conceptualization. **Firdausi Qadri:** Writing – review & editing, Validation, Supervision, Resources, Methodology, Investigation, Conceptualization. **Rachel A. McKendry:** Writing – review & editing, Validation, Supervision, Resources, Methodology, Investigation, Funding acquisition, Conceptualization. **Andrew S. Azman:** Writing – review & editing, Validation, Supervision, Resources, Methodology, Investigation, Conceptualization.

## Declaration of competing interest

The authors declare that they have no known competing financial interests or personal relationships that could have appeared to influence the work reported in this paper.

## Appendix A Supplementary data

Supplementary Data 1 Print time and costs for design files.
