# Supplementary figures and images for: Open Diagnostic Reader (ODR): An affordable, modular 3D-printed platform for standardized imaging and quantitative analysis of rapid diagnostic tests
Source: HardwareX. 2026 Jun 5;27:e00802. doi: 10.1016/j.ohx.2026.e00802 (PMC13272561; doi:10.1016/j.ohx.2026.e00802)

**Supplementary Tables**

**Supplementary Table 1.** Print time and costs for design files.


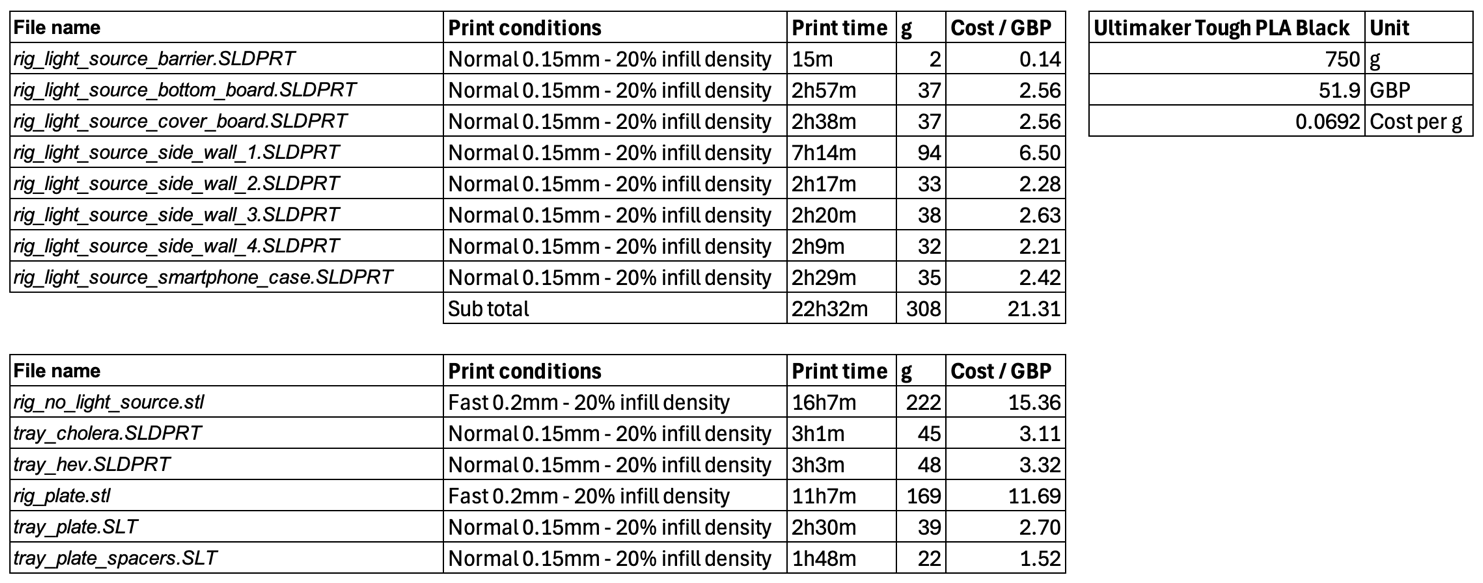

Supplement: Supplementary Data 1 — Print time and costs for design files. [file mmc1.docx]
